# Impact of prostate cancer screening in European ancestry un‐affected men with germline DNA repair pathogenic variants

**DOI:** 10.1002/bco2.424

**Published:** 2025-01-31

**Authors:** Vittorio Fasulo, Giuseppe Chiarelli, Giuseppe Garofano, Carla Barbara Ripamonti, Monica Barile, Paolo Bianchi, Emanuela Morenghi, Alessio Benetti, Muhannad Aljoulani, Alessio Finocchiaro, Marco Paciotti, Pier Paolo Avolio, Edoardo Beatrici, Paola Arena, Alberto Saita, Rodolfo Hurle, Federica Maura, Giorgio Da Rin, Rosanna Asselta, Anita Capalbo, Giulia Soldà, Paolo Casale, Nicolò Maria Buffi, Giovanni Lughezzani, Massimo Lazzeri

**Affiliations:** ^1^ Department of Biomedical Sciences Humanitas University Milan Italy; ^2^ Department of Urology IRCCS‐Humanitas Research Hospital Milan Italy; ^3^ Laboratory Analysis Unit IRCCS‐Humanitas Research Hospital Rozzano MI Italy; ^4^ Medical Genetics and RNA Biology Laboratory, IRCCS‐Humanitas Research Hospital Rozzano Milan Italy

**Keywords:** BRCA 1–2, DNA‐repair gene variant, genetic risk, prostatic neoplasm, screening

## Abstract

**Background and Objective:**

Prostate cancer (PCa) is a significant global health concern, ranking as the second most prevalent cancer among men worldwide. Genetic factors, particularly germline pathogenic variants (PVs) in DNA repair genes (DRGs), play a crucial role in PCa predisposition. Our study aimed to assess patients' adherence to a targeted PCa screening program targeting high‐risk individuals with DRG PVs and evaluate the potential reduction in biopsy and MRI rates by employing our screening protocol.

**Methods:**

We conducted a prospective ongoing trial evaluating targeted PCa screening in men with documented PVs in DRGs. Screening involved annual assessment of medical history, physical examination, prostate‐specific antigen (PSA) testing, Prostate Health Index (PHI), and multiparametric magnetic resonance imaging (mpMRI) when indicated. Descriptive statistics were used to analyse patient characteristics, and adherence to screening was evaluated at three time points: baseline (T0), one year (T1), and two years (T2) from enrolment.

**Key Findings and Limitations:**

A total of 101 high‐risk individuals were enrolled, with a median age of 52 years. Adherence to screening was high, with 72.3% of patients attending the first annual follow‐up (T1) and 100% attending the second follow‐up (T2). Despite elevated PSA levels in some patients, no PCa was detected during the study period. However, our screening protocol demonstrated the potential in reducing unnecessary biopsies and MRIs, particularly in patients with elevated PSA but low PHI values. Limitations include the ongoing nature of the study, small sample size, and lack of non‐carrier controls.

**Conclusions and Clinical Implications:**

Our findings described a new PCa screening strategy integrated with genetic risk factors. The incorporation of PHI shows promise in improving the efficiency of diagnostic procedures while minimizing unnecessary interventions. High adherence among high‐risk individuals underscores the potential effectiveness of targeted screening programs.

## 1 INTRODUCTION

Prostate cancer (PCa) ranks as the second most prevalent cancer among men globally. In 2020 alone, there were 1 414 259 reported cases of PCa, resulting in 375 304 deaths.^1^, ^2^ Among the key risk factors associated with PCa, a family history of the disease is paramount, with an estimated 5–10% of PCa cases attributed to inherited susceptibility.^3^ Notably, heterozygous germ‐line pathological variants (PVs) in tumour suppressor genes such as *BRCA2* significantly elevate PCa risk, particularly in men under 65 years old, and serve as an independent prognostic factor for poorer outcomes.^4^, ^5^

Research has revealed PVs in DNA‐repair genes (DRGs), including *BRCA1/BRCA2*, in approximately 8–12% of localized PCa cases and 20–25% of advanced metastatic castration‐resistant PCa cases.^6^, ^7^ These PVs, have significant implications for cancer diagnosis and treatment planning.^8^ Targeted screening of DRG PVs carriers may lead to early diagnosis, enhance prognosis and guide therapeutic decisions. The NCCN guidelines recommend prostate‐specific antigen (PSA) screening for men with *BRCA*1/2 PVs starting at age 40.^9^ The IMPACT study evaluates targeted PCa screening for men carrying genetic alterations in *BRCA1*, *BRCA2*, and Lynch syndrome genes (*MSH2*, *MSH6*, and *MLH1*), aiming to detect clinically significant PCa (csPCa) using a PSA threshold of 3.0 ng/ml.^10^, ^11^ Efforts such as the PRAISE‐U (Prostate Cancer Awareness and Initiative for Screening in the European Union) consortium seek to enable faster knowledge transfer and develop tailored, cost‐effective, risk‐based screening programs for PCa within the European Union.^12^ Recently, an algorithm for PCa screening in unaffected men with germline DRG PVs was presented, showcasing early results focused on enhanced screening empowered by the Prostate Health Index (PHI) and multiparametric magnetic resonance imaging (mpMRI).^13^

Based on this, our objective was to assess the patients' adherence to a targeted screening and the number of PCa cases detected within the screening program. Additionally, we aimed to evaluate the potential decrease in biopsy and/or MRI rates by utilizing our protocol.

## 2 MATERIALS AND METHODS

### 2.1 Study Design

This is a non‐profit charity‐funded prospective ongoing trial, designed to evaluate the clinical performance of targeted “enhanced” screening in men at high genetic risk for PCa because carriers of DRG PVs (AIRC ‐ Fondazione AIRC per la Ricerca sul Cancro project IG 2020 ID 25027). The study design was previously reported.^13^, ^14^ The study is conducted at a high‐volume single tertiary research hospital through the collaboration of the departments of Oncology, Breast Unit, Urology, Pathology, Radiology, and Medical Genetics. Patients were included if they met all the following inclusion criteria:

- Familiarity for breast/ovarian cancer in first‐degree female relatives (Eve's rib) or prostate cancer in male relatives (Adam's rib);
- A documented germline PV in a DRGs. Only variants predicted to be pathogenic or likely pathogenic according to the American College of Medical Genetics and Genomics (ACMG)‐recommended five variant classification categories were included (level 1 and 2 variants)^15^;
- Age between 35 and 69 years;
- The will to be compliant with the planned screening points.

Patients were excluded if they met any of the following criteria:

- Previous prostate endoscopic surgery;
- Previous diagnosis of PCa;
- Absolute and relative contraindications to MRI;
- Carriers of a variant of unknown significance (VUS, level 3 according to ACMG).

For selecting men with documented DRG PVs we used two strategies: Adam's rib and Eve's rib, as previously reported.^13^, ^14^, ^16^ Although VUS were excluded from the screening, relatives of patients from the Adams‐rib strategy who were found to have a VUS still underwent genetic counselling to evaluate the potential DRG PV. All the men who consented to participate underwent an annual targeted PCa screening, which consists of the collection of medical history, physical examination with digital rectal examination (DRE), a blood test measuring total PSA, free PSA and ‐2proPSA, and PHI, as well as mpMRI when indicated. Details were previously presented.^13^

### 2.2 Endpoints

The primary outcome was the patients' adherence to attending the targeted screening and the number of PCa detected. The secondary outcome was to evaluate the potential reduction in the rate of biopsies and/or MRIs by employing our protocol, which uses a PHI cut‐off of 20, instead of the previously evaluated trigger of PSA > 3 ng/ml.^10^

### 2.3 Statistical analysis

Descriptive statistics were calculated using frequency (N) and percentage (%) for categorical variables, while median and interquartile range (IQR) were used for continuous variables. We evaluated the screening path at three different times from the enrolment: time zero (T0, enrollement‐1° visit), one year (T1, 12 months‐2° visit), and two years (T2, 24 months‐3° visit). A box‐and‐whisker plot was used to display the distribution of PSA and PHI of all patients in follow‐up. We defined adherence as the patients' attendance at the follow‐up visit, given that they had already attended the visit the previous year. So, we evaluated the adherence at the annual follow‐up at T1 from T0, and at T2 from T1, by comparing the number of patients who attended the screening with those who participated in the visit one year prior.

We used descriptive statistics to analyse the patient cohort at various time points, we sought to explore the intersection of elevated PSA levels (>3 ng/ml) and PHI values, particularly focusing on the potential impact on the decision‐making process regarding MRI and biopsy.

Two‐sided *p* values <0.05 indicated statistical significance. All analyses were performed using Stata 17 (Stata Corp LLC, College Station, Texas, USA).

### 2.4 Role of the funding source

The study grant sponsor, AIRC ‐ Fondazione AIRC per la Ricerca sul Cancro, had no role in the data collection, data analysis, data interpretation, or writing of the report.

## 3 RESULTS

From January 2021 to July 2023 a total of 101 un‐affected carriers of a DRG PV accepted the targeted screening, with a median age of 52 (IQR 46–61). The majority were enrolled from Eve's rib strategy (94; 93.1%), and the remaining from Adam's rib strategy (7; 6.9%). Currently, we have screened 150 men for germline DRG PVs in those diagnosed with PCa (Adam's rib strategy). Of these, five had a DRG PV and 25 had a VUS, and we identified seven of their relatives with a DRG PV too, and all of them agreed to undergo the dedicated PCa screening.

As expected, PVs were most commonly found in *BRCA2* (56.4%), followed by *BRCA1* (19.8%), and *ATM* (4.0%). Additionally, 3.0% had PVs in PMS2, and 2.0% in MLH1 and MSH2. There were singular occurrences (1.0%) for BRIP1, MSH6, PALB2, BRCA3, and TMPRSS2‐ERG (Table 1).

**TABLE 1:** Percentage of pathogenetic variants (PV) in DRG for all 101 screened patients.

DRG PV	N (101)
*BRCA2*	57 (56.44%)
*BRCA1*	20 (19.80%)
*ATM*	4 (3.96%)
*PMS2*	3 (2.97%)
*BRIP1*	1 (0.99%)
*MLH1*	2 (1.98%)
*MSH2*	2 (1.98%)
*MSH6*	1 (0.99%)
*PALB2*	1 (0.99%)
*BRCA3*	1 (0.99%)
TMPRSS2‐ERG	1 (0.99%)
Missing	8 (7.92%)

A total of 101 individuals were enrolled at stage T0. Among these, a total of 58 individuals had a PHI <20, with only 1 (1.70%) of them undergoing a mpMRI (for positive DRE). A total of 27 individuals had a PHI 20–40, with 22 (81.5%) undergoing a mpMRI. Only three individuals had PHI ≥40, with two of them (66.7%) undergoing a mpMRI. Among the 88 patients who underwent a PHI test, the median total PSA was 0.9 (IQR 0.5–1.65) ng/ml, proPSA was 4.35 (IQR 2.65–7.25) ng/ml, and PHI was 17 (IQR 12–24) ng/ml.

At the first annual follow‐up (T1), a total of 73 individuals attended the annual screening. Among these, 48 individuals had a PHI < 20, with only two (4.2%) of them undergoing a mpMRI (for positive DRE). A total of 19 individuals had a PHI 20–40, with seven (36.8%) undergoing a mpMRI. Only three individuals had PHI ≥40, with two of them (66.7%) undergoing a mpMRI. Among the 70 patients who underwent a PHI test, the median total PSA was 0.82 (IQR 0.5–1.5) ng/ml, Pro PSA was 4.59 (IQR 3.14–8.06) ng/ml, and PHI was 18 (IQR 14–23) ng/ml.

**FIGURE 1:**
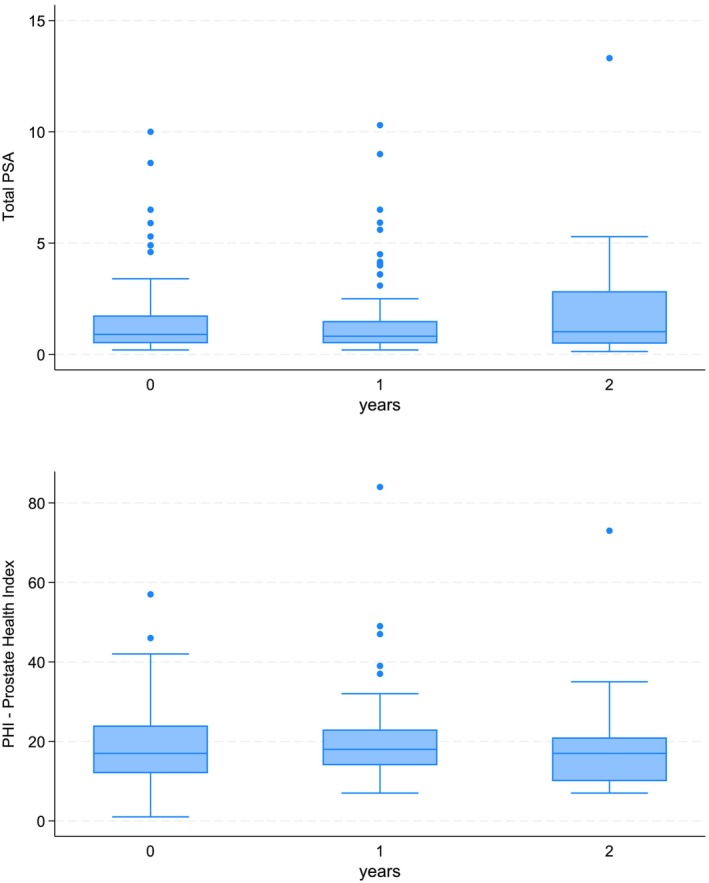
The box‐and‐whisker plot below displays the distribution of PSA and PHI at three different time points: T0 (baseline enrolment time), T1 (annual follow‐up at 1 year), and T2 (annual follow‐up at 2 years). No statistical significance was found for either PSA (*p* = 0.325) or PHI (*p* = 0.889).

At the second follow‐up assessment (T2), a total of 33 individuals attended the annual screening. Among these, 22 individuals had a PHI < 20, with only one (4.6%) of them undergoing a mpMRI (for suspected DRE). A total of eight individuals had a PHI 20–40, with two (25.0%) undergoing a mpMRI, while only one individual had PHI ≥40, and he did not undergo a mpMRI. Notably, the majority of visits and lab tests are ongoing, but among those available, the median total PSA was 1.02 (IQR 0.48–2.84) ng/ml, Pro PSA was 4.47 (IQR 2.66–7.47) ng/ml, and PHI was 17 (IQR 10–21) ng/ml. The distribution of PSA and PHI at the three different time‐points (T0, T1 and T2) is shown in Figures 1. Their values are reported in Table 2.

**TABLE 2:** The table below shows median values of total PSA, Pro PSA, and PHI at the time of T0 (enrolment ‐ 1st visit), T1 (12 months ‐ 2nd visit), and T2 (24 months ‐ 3rd visit), expressed as median (IQR).

T0	Median (IQR)
Total PSA *(ng/ml)*	0.9 (0.5–1.65)
Pro PSA *(ng/ml)*	4.35 (2.65–7.25)
PHI *(ng/ml)*	17 (12–24)
Missing values	13
T1	
Months from enrolment	12 (12–13)
Total PSA *(ng/ml)*	0.82 (0.5–1.5)
Pro PSA *(ng/ml)*	4.59 (3.14–8.06)
PHI *(ng/ml)*	18 (14–23)
T2	
Months from enrolment	24 (24–25)
Total PSA *(ng/ml)*	1.02 (0.48–2.84)
Pro PSA *(ng/ml)*	4.47 (2.66–7.47)
PHI *(ng/ml)*	17 (10–21)

Regarding the patients' adherence at screening, at the first annual follow‐up (T1), of the 101 patients enrolled at baseline (T0), 73 (72.3%) underwent a re‐evaluation. At the second annual follow‐up (T2), out of the 33 patients who were expected at that time point for the second evaluation all of them (33; 100%) accepted to be re‐examined.

Evaluating the potential reduction in the rate of biopsies and/or MRIs by employing our screening protocol, at T0 among 12 patients with PSA > 3 ng/ml, none had a PHI <20, indicating a potential alignment with conventional screening that recommends further investigation for this subgroup. By T1, this consistency remained with 12 patients with PSA > 3 ng/ml and PHI >20. However, at T2, two out of six patients had both PSA > 3 ng/ml and PHI <20, and they were managed to avoid the recommended mpMRI and eventual biopsy according to IMPACT criteria.^10^

We performed 6 biopsies for suspected PCa, all of which were negative. Five were conducted because PHI was ≥40, and one because mpMRI reported a PI‐RADS ≥3. However, during the follow‐up, a patient was diagnosed with a bladder tumour.

## 4 DISCUSSION

We presented the preliminary results of our targeted screening for a high‐risk cohort of unaffected men of European ancestry with a DRG PV.

Being un‐affected men with a pathogenic or pathogenic‐like germline variant in a DRG is associated with a higher risk of PCa, especially among *BRCA2* mutation carriers (OR = 2.64, 95%CI:2.03–3.47), and is also associated with poorer cancer‐specific (CSS; HR = 2.53, 95%CI:1.98–3.22) and overall survival rates (HR 2.08, 95%CI:1.55–2.79).^17^ Bancroft et al. reported a significantly higher incidence of PCa in men with PVs in *MSH2* and *MSH6* compared with non‐carrier controls, supporting an increased risk of PCa in individuals with Lynch syndrome.^18^ These findings support the use of targeted screening in men with DRG PV to detect csPCa. Nevertheless, the risk‐adapted screening strategy has its challenges, including “age” of intervention, the avoidance of over‐diagnosis and overtreatment, and a need for an appropriate knowledge of PCa genetics.

The median age at entry in our cohort was lower compared with previous studies (52 years, range 46–61). D'Elia et al. reported a mean age of PCa onset of 67.4 years in germline mutated Italian patients and 68.3 years in non‐mutated patients.^19^ Page et al. reported a median enrolment age of 54 years, but their cohort included carriers and non‐carriers of *BRCA1/2* PVs.^5^ Carriers of *MSH2* and *MSH6* PVs had mean age of 51.9 years and 53.6 years respectively. In our study we selected men with a PV in a DRG and age between 35 and 69 years. They were selected mainly as relatives of female probands with HBOC, while only a few from the Adams line (7%).^13^ This is attributed to the earlier initiation of DRG PV assessment within the context of breast and ovarian cancer at our institution, thus allowing us to recruit initially more individuals among the relatives of patients with HBOC. Our enrolment criteria may facilitate early diagnosis, however, the low median age at enrolment may account for the absence of any detected PCa thus far.

Our screening protocol would aim to diagnose csPCa, missing indolent PCa, avoiding overdiagnosis and over treatment. The PRACTICAL consortium investigated a pooled cohort of germline *ATM* PV carriers, concluding that although they were associated with younger age of PCa onset, variants did not conclusively predispose carriers to more aggressive PCa, and *BRCA2* is the only gene in which PVs have consistently been linked to aggressive PCa.^20^ The IMPACT study showed a median PSA 0.9 ng/ml at the time of enrolment in both *BRCA1* and *BRCA2* PV carriers, and a PSA > 3 ng/ml in 7.5% and 7.9%, respectively. Biopsies were performed in 82% of *BRCA2* subset and 78% of *BRCA1*, with a PCa detection rate of 2.8% and 2.6%, respectively. The median age at PCa diagnosis was 61 year (IQR: 56–64) in *BRCA2* and 23% were non‐csPCa.^5^ Our study strategy, aimed at avoiding the diagnosis of non‐csPCa, using a different cut‐point for mpMRI and/or biopsy, may be one of the reasons why we did not find any PCa after two years and half of follow‐up.

We observed that among patients with a PSA > 3 ng/ml, those with a PHI <20 demonstrated a significantly lower likelihood of undergoing further procedures compared with those with a higher PHI value. Notably, this reduction in unnecessary procedures was particularly evident in two out of six patients at the second follow‐up, where they had both a PSA > 3 ng/ml and a PHI <20, and following our protocol they avoided mpMRI and eventual biopsy, as would have been according to the IMPACT criteria. Our results indicate that the introduction of the PHI as an additional tool for PCa screening may allow for more accurate selection of patients who may benefit from further diagnostic procedures. These findings highlight the potential utility of PHI in reducing unnecessary imaging and biopsies, thereby minimizing patient discomfort and healthcare costs while ensuring appropriate management of patients with a suspected PCa.

The American and European Urological Associations recommend mpMRI before prostate biopsy. Moreover, the PROSTAGRAM trial reported that mpMRI was better than PSA in avoiding biopsy and increasing detection of ISUP grade>1, with depletion of over‐diagnosed indolent PCa.^21^ Minor evidence exists for men with a genetic risk of PCa. Segal et al. recruited 188 men (108 *BRCA1*, 80 *BRCA2*), and reported that 36% had a suspicious lesion on mpMRI and 8.5% were diagnosed with PCa; 44% of the tumours were classified as intermediate‐ or high‐risk diseases. mpMRI‐based screening missed only one of the cancers (6%), while age‐stratified PSA would have missed five (31%). Decision curve analysis showed that mpMRI screening, regardless of PSA level, had the highest net benefit for PCa diagnosis, especially in men aged <55 years.^22^ Cussenott et al. reported that using a mpMRI PI‐RADS score≥3 as the threshold, the 44% of prostate biopsies could be avoided, while missing a few csPCas. According to the authors, the screening of men at high genetic risk of PCa must be based on mpMRI without pre‐screening based on a PSA level of >3 ng/ml, to avoid missing too many ISUP grade >1 tumours and to significantly reduce the number of unnecessary biopsies.^23^ The protocol of our screening based on including mpMRI, aimed to reduce the rate of non‐csPCa, but so far, we cannot draw any significant conclusion.

Of note, at T2 all the patients we expected to follow up to that point, 100% continued with the screening by attending the second visit. Our preliminary findings provide us a rate of patients' adherence to the screening, which is between 72.3% (T1) and 100% (T2). We justify the lower rate at T1 as, when the enrolment started, we initially considered all available patients. However, on the first visit, many delayed because of COVID‐19 pandemic or logistical reasons, such as being out of the region or abroad.

Our study has limitations, including a small sample size hindering the assessment of PCa detection and the absence of non‐carrier controls. In addition, the incomplete follow‐up at T2 is another limitation. Furthermore, it is limited to European ancestry un‐affected cases and no cost‐effectiveness analysis was provided. Oh et al. investigated the value of knowing BRCA information in a potential screening plan and provided initial evidence regarding the cost‐effectiveness of genetically testing patients for *BRCA* mutations and treating them accordingly.^24^ We did not investigate the impact of genetic analysis cascade (family history, counselling and testing) on anxiety, depression, and cancer worry levels among probands and unaffected men enrolled in the screening program.^25^ Other limitations are that we did not consider any other biomarker, as PSA density, or urine marker, or another genetic markers such as TMPRSS2‐ERG fusion.^26^

## 5 CONCLUSION

Our findings describe the potential role of incorporating genetic and/or epidemiological risk factors may have within a prostate cancer screening strategies. Further research is warranted to evaluate the long‐term impact of these screening approaches, including the need for longer follow‐up periods, larger sample sizes, and international multicentric studies to validate our findings.

## AUTHOR CONTRIBUTIONS

*Conception and design*: Massimo Lazzeri. *Acquisition of data*: Nicolò Maria Buffi, Giovanni Lughezzani, Massimo Lazzeri, Giuseppe Garofano, Monica Barile, Carla Barbara Ripamonti, Paolo Bianchi, Alessio Benetti, Marco Paciotti, Muhannad Aljoulani, Alessio Finocchiaro, Paolo Arena, Edoardo Beatrici, Pier Paolo Avolio, Alberto Saita, Rodolfo Hurle, Federica Maura, Giorgio Da Rin, Anita Capalbo, Rosanna Asselta, and Giulia Soldà. *Analysis and interpretation of data*: Vittorio Fasulo, Giuseppe Chiarelli, and Massimo Lazzeri. *Drafting of the manuscript*: Vittorio Fasulo, Giuseppe Chiarelli, and Massimo Lazzeri. *Critical revision of the manuscript for important intellectual content*: All authors. *Statistical analysis*: Vittorio Fasulo, Giuseppe Chiarelli, Massimo Lazzeri, and Emanueala Morenghi. *Obtaining funding*: Massimo Lazzeri and Nicolò Maria Buffi. *Supervision*: Paolo Casale and Rosanna Asselta.

## CONFLICT OF INTEREST STATEMENT

None of the authors have any relevant disclosures, and none of the authors have any financial or non‐financial interests that may be relevant to the submitted work.
